# Registered Trials on Artificial Intelligence Conducted in Emergency Department and Intensive Care Unit: A Cross-Sectional Study on ClinicalTrials.gov

**DOI:** 10.3389/fmed.2021.634197

**Published:** 2021-03-24

**Authors:** Guina Liu, Nian Li, Lingmin Chen, Yi Yang, Yonggang Zhang

**Affiliations:** ^1^Department of Periodical Press and National Clinical Research Center for Geriatrics, West China Hospital, Sichuan University, Chengdu, China; ^2^West China School of Medicine, Sichuan University, Chengdu, China; ^3^Department of Medical Administration, West China Hospital, Sichuan University, Chengdu, China; ^4^Department of Anesthesiology and National Clinical Research Center for Geriatrics, West China Hospital, Sichuan University and The Research Units of West China (2018RU012), Chinese Academy of Medical Sciences, Chengdu, China; ^5^Department of Clinical Medicine, Gansu University of Traditional Chinese Medicine, Lanzhou, China; ^6^Chinese Evidence-based Medicine Center, West China Hospital, Sichuan University, Chengdu, China; ^7^Nursing Key Laboratory of Sichuan Province, Chengdu, China

**Keywords:** artificial intelligence, emergency department, intensive care unit, ClinicalTrials.gov, cross-sectional, trial

## Abstract

**Objective:** Clinical trials contribute to the development of clinical practice. However, little is known about the current status of trials on artificial intelligence (AI) conducted in emergency department and intensive care unit. The objective of the study was to provide a comprehensive analysis of registered trials in such field based on ClinicalTrials.gov.

**Methods:** Registered trials on AI conducted in emergency department and intensive care unit were searched on ClinicalTrials.gov up to 12th January 2021. The characteristics were analyzed using SPSS21.0 software.

**Results:** A total of 146 registered trials were identified, including 61 in emergency department and 85 in intensive care unit. They were registered from 2004 to 2021. Regarding locations, 58 were conducted in Europe, 58 in America, 9 in Asia, 4 in Australia, and 17 did not report locations. The enrollment of participants was from 0 to 18,000,000, with a median of 233. Universities were the primary sponsors, which accounted for 43.15%, followed by hospitals (35.62%), and industries/companies (9.59%). Regarding study designs, 85 trials were interventional trials, while 61 were observational trials. Of the 85 interventional trials, 15.29% were for diagnosis and 38.82% for treatment; of the 84 observational trials, 42 were prospective, 14 were retrospective, 2 were cross-sectional, 2 did not report clear information and 1 was unknown. Regarding the trials' results, 69 trials had been completed, while only 10 had available results on ClinicalTrials.gov.

**Conclusions:** Our study suggest that more AI trials are needed in emergency department and intensive care unit and sponsors are encouraged to report the results.

## Introduction

Artificial intelligence (AI), described as the science and engineering of making intelligent machines (1), is a broad term that implies the use of a computer to model intelligent behavior with minimal human intervention, generally at a speed and scale that exceed human capability (2–5). With the achievement of computer science, AI is involved in clinical practice, including tracking data (6, 7), diagnosis (8), and support of decision making (9, 10). AI has been widely used in clinical practices, such as in prediction, decision support, and the delivery of personalized health care (11–13), especially in diagnosis and treatment of acute events (14) to improve outcomes (15–17).

Emergency and critical care focus on resuscitating unstable patients and allowing time for recovery or the effect of specific therapies (18), and it can be provided in emergency department (ED) or intensive care unit (ICU) (18, 19). Emergency and critical care can be affected by levels of staffs, equipment and knowledge (18, 20). Adverse emergency and critical care will result in burdens and adverse outcomes, including weakness, dysfunction, contractures, pain, depression, anxiety, post-traumatic stress disorder, and even death (21–23). Early and fast diagnosis could save lives. Thus, using AI tools to fastly and accurately diagnostic will help a lot (10), especially to assist in uncertainty (24) or to further developing strategies (25). Will AI tools help physicians or patients in ED and ICU (26), there is still limited information and it should be assessed by well-deigned trials.

Well-designed trials can assist clinical practice (27, 28) and transparency is the key characteristic for well-designed trials. Pre-registered in public registries is the most important strategy to ensure transparency (29) and now been required for all trials by The International Committee of Medical Journal Editors (ICMJE). Thus, analyzing registered trials will know the progress in such field, and many studies have been published to analyze registered trials in Clinicaltrials.gov, such as acupuncture (30), ventilator-associated pneumonia (VAP) (31), old populations with infectious diseases (32), and cancer diagnosis (33). However, there is no such study for AI in ED and ICU. Thus, we conducted the current study to provide a comprehensive analysis of the development of AI for ED and ICU.

## Materials and Methods

### Reporting Guideline

This is a cross-sectional study, and it was reported according to STROBE (34).

### Data Source

A cross-sectional study about registered trials for AI in ED and ICU on ClinicalTrials.gov was carried out, and the searched words were as follows: artificial intelligence, AI, computational intelligence, machine intelligence, machine learning, deep learning, algorithms, computer reasoning, computer vision system, knowledge acquisition (computer), knowledge representation (computer), natural language processing, neural networks of computer, robotics. All information was downloaded, and duplicates were removed by Excel (Office 365, Microsoft, Redmond, WA, USA) according to the trials' national clinical trial (NCT) number.

### Data Selection and Eligible Trials

We selected trials mainly according to their conditions or study descriptions. Inclusion criteria: Trials on AI and only conducted at ED and ICU. Exclusion criteria: trials not related to artificial intelligence; trials excluded conditions in the ED or ICU; trials conducted in general wards.

### Studied Variables

The studied variables included study type, start year, enrollment, participant age, participant gender, status, phase, study results, sponsor, main funding source, number of funding sources, location, number of centers, primary purpose, intervention, allocation, intervention model, masking, observational model, and time perspective.

### Statistical Analysis

The characteristics were analyzed by descriptive methods. The continuous variables were characterized as median and interquartile ranges (IQR), and the categorical variables were reported as frequencies and percentages. The study types included interventional trials and observational trials. The start year was when the trial was first posted on ClinicalTrial.gov, including 2004–2010, 2011–2016, and 2017–2021. Whether the results were available or unavailable was also analyzed. The sponsor included university, hospital, industry/company, or others, including individuals, institutions, or some organizations that cannot be included in other categories. The main funding resources included industry, the federal reserve of United States (U.S. fed), or other resources, such as universities, individuals, and organizations that cannot be divided into subtypes. Data analysis was performed using SPSS21.0 software.

## Results

### Basic Characteristics

**Figure 1:**
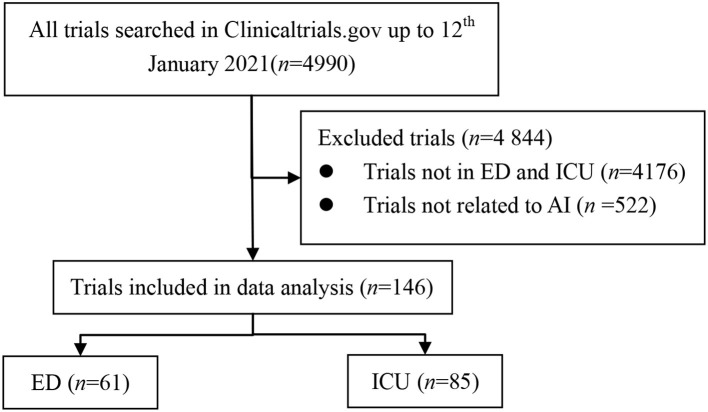
Flowchart of recruited trials registered in ClinicalTrials.gov up to 12th January 2021.

Up to 12th January 2021, 4990 trials were identified after the initial search. After reviewing all information, a total of 146 registered trials were included (Figure 1). The characteristics of the included trials are shown in Table 1. Among the 146 trials, 85 (58.22%) were interventional trials, and 61 (41.78%) were observational trials. Seventy-five (51.37%) trials registered after 2017, while 25 (17.12%) and 46 (31.51%) registered in 2004–2010 and 2011–2016, respectively. Sample sizes were from 0 to 18,000,000, with a median of 233. For genders, 143 (97.95%) trials recruited both male and female participants; however, three trials (2.05%) recruited females only. For age, 112 (76.71%) trials only recruited adults, 11 (7.53%) only recruited children, while 23 (15.75%) recruited both adults and children. For status, 23 (15.75%) trials were not yet recruiting, 30 (20.55%) were recruiting, 69 (47.26%) were completed, 1 was suspended, 10 were terminated or withdrawn and 13 were in unknown status. For results, only 10 (6.85%) trials reported results on ClinicalTrials.gov, while 136 (93.15%) did not report results. For sponsors, 63 (43.15%) were sponsored by universities, 52 (35.62%) were sponsored by hospitals, 14 (9.59%) were sponsored by industries/companies, and 17 (11.64%) were sponsored by other institutions. For funding, 15 (10.27%) were funded by industries, and 131 (89.73%) did not report clear funding sources. For locations, 58 (39.73%) trials were conducted in America, 58 (39.73%) in Europe, 9 (6.16%) in Asia, 4 (2.74%) in Australia, and 17 (11.64%) did not report locations.

**Table 1:** The characteristics of the 146 trials registered on ClinicalTrial.gov.

**Characteristics**		**Number**	**Percentage (%)**
**Study type**			
	Interventional	85	58.22
	Observational	61	41.78
**Registered year**			
	2004–2010	25	17.12
	2011–2016	46	31.51
	2017–2021	75	51.37
**Enrollment**			
	0–100	53	36.30
	100–500	47	32.19
	>500	45	30.82
	Unknown	1	0.68
**Gender**			
	Female only	3	2.05
	Both	143	97.95
**Participant age (year)**			
	<18	11	7.53
	≥18	112	76.71
	Both	23	15.75
**Status**			
	Not recruiting	23	15.75
	Recruiting	30	20.55
	Completed	69	47.26
	Suspended	1	0.68
	Terminated/withdrawn	10	6.85
	Unknown	13	8.91
**Study results**			
	Has results	10	6.85
	No results available	136	93.15
**Sponsor**			
	University	63	43.15
	Hospital	52	35.62
	Industry/company	14	9.59
	Other	17	11.64
**Funding source**			
	Industry	15	10.27
	Other	131	89.73
**Location**			
	America	58	39.73
	Europe	58	39.73
	Asia	9	6.16
	Australia	4	2.74
	Unknown	17	11.64

### Characteristics of Study Design

#### Interventional Study

The characteristics of the 85 interventional studies are shown in Table 2. Thirteen (15.29%) trials were for diagnosis, 33 (38.82%) for treatment, 16 (18.82%) for prevention, 15 (17.65%) for supportive care, 6 (7.06%) for health services research and 2 (2.35%) did not report the clear purpose. Twety-one (24.71%) trials were for behavioral intervention, 28 (32.94%) for intervention device, 6 (7.06%) for diagnostic test, 7 (8.24%) for the procedure and 23 (27.06%) did not have clear information on intervention. For the types of assignments, 53 (62.35%) were parallel assignment, 24 (28.24%) were single group assignment, 1 (1.18%) was factorial assignment, 3(3.53%) were crossover assignment, 3(3.6%) were sequential assignment and 1(1.2%) was unknown, respectively. For allocation, 59 (69.41%) were randomized, 11 (12.94%) were nonrandomized, 14 (16.47%) were not applicable and 1 (1.18%) was unknown. For masking, 52 (61.18%) were open-labeled, 20 (23.53%) were single-masked, 8 (9.41%) were double-masked, 2(2.35%) were triple-masked, 2(2.35%) were quadruple-masked and 1 (1.18%) had no information. For sample size, 24 (28.23%) trials recruited more than 500 participants, while 39 (45.88%) recruited <100 participants and 22 (25.88%) recruited 100–500 participants. For gender, 1 (1.18%) trial included female only and 84 (98.82%) recruited both male and female. For age, 63 (74.12%) trials recruited adult only, while 8 (9.41%) trials recruited child only and 14 (16.47%) trials recruited both child and adult. One (1.18%) trial was in phase 2, 1 (1.18%) in phase 2/3, 3(3.53%) in phase 3, 1 (1.18%) in phase 4 and 79 had no clear information. For status, 46 (54.12%) trials were completed, 15 (17.65%) were recruiting, 9 (10.59%) were not recruiting, 7 (8.24%) were terminated or withdrawn, 1 (1.18%) was suspended and 7 (8.24%) had no information. Among all 85 interventional trials, only 10 trials reported results on Clinicaltrials.gov. For sponsors, 43 (50.59%) were sponsored by universities, 25 (29.41%) were sponsored by hospitals, 8 (9.41%) were sponsored by industries/companies, and 9 (10.59) were sponsored by other institutions. For funding, 8 (9.41%) trials were funded by industries and 77 (90.59%) did not report funding sources. For locations, 42 (49.41%) were from America, 28 (32.94%) were from Europe, 4 (4.71%) were from Asia, 4 (4.71%) were from Australia and 7 (8.24%) did not report location information.

**Table 2:** Designs of 85 interventional trials registered with ClinicalTrial.gov.

**Characteristics**		**Number**	**Percentage (%)**
**Primary purpose**			
	Diagnosis	13	15.29
	Treatment	33	38.82
	Prevention	16	18.82
	Supportive care	15	17.65
	Health services research	6	7.06
	Unknown	2	2.35
**Intervention**			
	Behavioral	21	24.71
	Device	28	32.94
	Diagnostic test	6	7.06
	Procedure	7	8.24
	Other	23	27.06
**Intervention model**			
	Parallel assignment	53	62.35
	Factorial assignment	1	1.18
	Crossover assignment	3	3.53
	Single group assignment	24	28.24
	Sequential assignment	3	3.53
	Unknown	1	1.18
**Allocation**			
	Randomized	59	69.41
	Nonrandomized	11	12.94
	N/A	14	16.47
	Unknown	1	1.18
**Masking**			
	Single	20	23.53
	Double	8	9.41
	Triple	2	2.35
	Quadruple	2	2.35
	None (open-label)	52	61.18
	Unknown	1	1.18
**Enrollment**			
	0–100	39	45.88
	100–500	22	25.88
	>500	24	28.23
**Gender**			
	Both	84	98.82
	Female	1	1.18
**Participant age (year)**			
	<18	8	9.41
	≥18	63	74.12
	Both	14	16.47
**Status**			
	Not recruiting	9	10.59
	Recruiting	15	17.65
	Completed	46	54.12
	Suspended	1	1.18
	Terminated/withdrawn	7	8.24
	Unknown	7	8.24
**Results**			
	Has results	10	11.76
	No results available	75	88.24
**Sponsor**			
	University	43	50.59
	Hospital	25	29.41
	Industry/company	8	9.41
	Other	9	10.59
**Funding source**			
	Industry	8	9.41
	Other	77	90.59
**Location**			
	America	42	49.41
	Europe	28	32.94
	Asia	4	4.71
	Australia	4	4.71
	Unknown	7	8.24

#### Observational Study

The characteristics of the 61 observational studies are shown in Table 3. Among them, 35 (57.38%) were cohort studies, 9 (14.75%) were case-only studies, 8 (13.11%) were case-control studies and one was case-crossover study, while 6 (9.84%) had no clear information and 2 (3.28%) did not provide information. Forty-two (68.85%) were prospective studies, 14 (22.95%) were retrospective studies, 2 (3.28%) were cross-sectional studies, 2 (3.28%) were other designed studies and one did not report related information. For sample size, 21 (34.43%) recruited more than 500 participants, while 14 (22.95%) recruited <100 participants and 25 (40.98%) recruited 100–500 participants. For gender, only 2 studies included female only and 59 (96.72%) recruited both male and female. For age, 49 (80.33%) recruited adult only, while 3 (4.92%) recruited child only and 9 (14.75%) recruited both child and adult. For status, 23 (37.70%) were completed, 15 (24.59%) were recruiting, 14 (22.95%) were not recruiting, 3 (4.92%) were terminated or withdrawn and 6 (9.84%) had no information. Among all 61 observational studies, none of them reported results on Clinicaltrials.gov. For sponsors, 20 (32.79%) were sponsored by universities, 27 (44.26%) were sponsored by hospitals, 6 (9.84%) were sponsored by industries/companies, and 8 (13.11%) were sponsored by other institutions. For funding, 7 (11.48%) were funded by industries, and 54 (88.52%) did not report clear funding sources. For locations, 30 (49.18%) were from Europe, 16 (26.23%) were from America, 5 (8.20%) were from Asia and 10 (16.39%) did not report locations.

**Table 3:** Designs of 61 observational trials registered on ClinicalTrial.gov.

**Characteristics**		**Number**	**Percentage (%)**
**Observational model**			
	Case-control	8	13.11
	Case-only	9	14.75
	Case-crossover	1	1.64
	Cohort	35	57.38
	Other	6	9.84
	Unknown	2	3.28
**Time perspective**			
	Prospective	42	68.85
	Retrospective	14	22.95
	Cross-sectional	2	3.28
	Other	2	3.28
	Unknown	1	1.64
**Enrollment**			
	0–100	14	22.95
	100–500	25	40.98
	>500	21	34.43
	Unknown	1	1.64
**Participant gender**			
	Female only	2	3.28
	Both	59	96.72
**Participant age (year)**			
	<18	3	4.92
	≥18	49	80.33
	Both	9	14.75
**Status**			
	Not recruiting	14	22.95
	Recruiting	15	24.59
	Completed	23	37.70
	Terminated/withdrawn	3	4.92
	Unknown	6	9.84
**Results**			
	Has results	0	0.00
	No results available	61	100.00
**Sponsor**			
	University	20	32.79
	Hospital	27	44.26
	Industry/company	6	9.84
	Other	8	13.11
**Funding source**			
	Industry	7	11.48
	Other	54	88.52
**Location**			
	America	16	26.23
	Europe	30	49.18
	Asia	5	8.20
	Unknown	10	16.39

### Characteristics of Trials at Emergency Department

Table 4 shows the characteristics of trials conducted in ED. Among the 61 trials, 37 (60.66%) were interventional trials, and 24 (39.34%) were observational trials. Thirty-four (55.73%) trials registered after 2017, while 8 (13.11%) and 19 (31.15%) were registered in 2004–2010 and 2011–2016, respectively. For sample size, 27 (44.26%) trials recruited more than 500 participants, while 14 (22.95%) recruited <100 participants and 20 (32.79%) recruited 100 to 500 participants. For genders, 60 trials (98.36%) recruited both male and female participants; however, 1 (1.64%) recruited females only. For age, 39 trials (63.93%) only recruited adults, 6 (9.84%) only recruited children, while 16 (26.23%) recruited both adults and children. For status, 9 (14.75%) were not yet recruiting, 10 (16.39%) were recruiting, 30 (49.18%) were completed, six were terminated or withdrawn and six were in unknown status. For results, only three trials reported results on Clinicaltrials.gov, while 58 (95.08%) did not report results. For sponsors, 28 (45.90%) were sponsored by universities, 25 (40.98%) were sponsored by hospitals, 4 (6.56%) were sponsored by industries/companies, and 4 (6.56%) were sponsored by other institutions. For funding, 4 trials (6.56%) were funded by industries and 57 (93.44%) did not report clear funding sources. For locations, 28 (45.90%) were in America, 26 (42.62%) in Europe, 1 (1.64%) in Asia and 6 (9.84%) did not report locations.

**Table 4:** The characteristics of the 61 trials in ED registered on ClinicalTrial.gov.

**Characteristics**		**Number**	**Percentage (%)**
**Study type**			
	Interventional	37	60.66
	Observational	24	39.34
**Start year**			
	2004–2010	8	13.11
	2011–2016	19	31.15
	2017–2021	34	55.73
**Enrollment**			
	0–100	14	22.95
	100–500	20	32.79
	>500	27	44.26
**Gender**			
	Female only	1	1.64
	Both	60	98.36
**Participant age (year)**			
	<18	6	9.84
	≥18	39	63.93
	Both	16	26.23
**Status**			
	Not recruiting	9	14.75
	Recruiting	10	16.39
	Completed	30	49.18
	Terminated/withdrawn	6	9.84
	Unknown	6	9.84
**Study results**			
	Has results	3	4.92
	No results available	58	95.08
**Sponsor**			
	University	28	45.90
	Hospital	25	40.98
	Industry/company	4	6.56
	Other	4	6.56
**Funding source**			
	Industry	4	6.56
	Other	57	93.44
**Location**			
	America	28	45.90
	Europe	26	42.62
	Asia	1	1.64
	Unknown	6	9.84

### Characteristics of Trials at ICU

Table 5 shows the characteristics of trials on AI conducted in emergency department. Among the 85 trials, 48 (56.47%) were interventional trials, and 37 (43.53%) were observational trials. Forty-one (48.24%) trials registered after 2017, while 17 (20.00%) and 27 (31.76%) registered in 2004–2010 and 2011–2016, respectively. For sample size, 18 (21.18%) trials recruited more than 500 participants, 39 (45.88%) recruited <100 participants, 27 (31.76%) recruited 100–500 participants and 1 was unknown. For genders, 83 trials (97.65%) recruited both male and female participants; however, 2 (2.35%) trials recruited females only. For age, 73 trials (85.88%) only recruited adults, 5 (5.88%) trials only recruited children, while 7 (8.24%) recruited both adults and children. For status, 14 (16.47%) were not yet recruiting, 20 (23.53%) were recruiting, 39 (45.88%) were completed, while one was suspended, four were terminated or withdrawn and seven were in unknown status. For results, only seven trials reported results on Clinicaltrials.gov, while 78 (91.76%) did not report results. For sponsors, 35 (41.18%) trials were sponsored by universities, 27 (31.76%) were sponsored by hospitals, 10 (11.76%) were sponsored by industries/companies, and 13 (15.29%) were sponsored by other institutions. For funding, 11 trials (12.94%) were funded by industries and 74 (87.06%) did not report clear funding sources. For locations, 30 (35.29%) were in America, 32 (37.65%) were in Europe, 8 (9.41%) in Asia, 4 (4.71%) in Australia and 11 (12.94%) did not report locations.

**Table 5:** The characteristics of the 85 trials in ICU registered on ClinicalTrial.gov.

**Characteristics**		**Number**	**Percentage (%)**
**Study type**			
	Interventional	48	56.47
	Observational	37	43.53
**Start year**			
	2004–2010	17	20.00
	2011–2016	27	31.76
	2017–2021	41	48.24
**Enrollment**			
	0–100	39	45.88
	100–500	27	31.76
	>500	18	21.18
	Unknown	1	1.18
**Gender**			
	Female only	2	2.35
	Both	83	97.65
**Participant age (year)**			
	<18	5	5.88
	≥18	73	85.88
	Both	7	8.24
**Status**			
	Not recruiting	14	16.47
	Recruiting	20	23.53
	Completed	39	45.88
	Suspended	1	1.18
	Terminated/withdrawn	4	4.71
	Unknown	7	8.24
**Study results**			
	Has results	7	8.24
	No results available	78	91.76
**Sponsor**			
	University	35	41.18
	Hospital	27	31.76
	Industry/company	10	11.76
	Other	13	15.29
**Funding source**			
	Industry	11	12.94
	Other	74	87.06
**Location**			
	America	30	35.29
	Europe	32	37.65
	Asia	8	9.41
	Australia	4	4.71
	Unknown	11	12.94

## Discussion

Clinical trials have played important roles in changing clinical practice (19, 35, 36). Analyzing registered trials could provide a comprehensive analysis of progress in a specific field; thus, numerous studies have been published to analyze registered trials on Clinicaltrials.gov. Considering AI is important tool and have been applied in ED and ICU, we performed the current study to analyze registered trials on AI conducted in ED and ICU.

A total of 146 registered trials were identified, including 61 trials in ED and 85 in ICU, which is similar with our previous study for cancer (33). Over half trials registered after 2017, and it was consistent with the development of industry 4.0, which depended on AI to empower medicine (37). Research in children was often challenging due to scientific, ethical, and practical factors, so only 23.29% trials enrolled children, and 17% enrolled children from 2007 to 2010 (38). More work is needed to ensure that children are equally involved in trials on AI in ED and ICU. In our study, most registered trials included relatively large samples, which would help to reduce the potential risk of statistical error (39). It is interesting to know that no trials were funded by NIH, which did not mean NIH did not fund trials in such field, because academic institutions/medical centers might have been funded by NIH to perform the trials, and they did not report it clearly in the website of Clinicaltrials.gov (30).

Reporting trials' results is very important. In our study, 47.26% trials had been completed, but only 6.85% reported results on ClinicalTrials.gov, suggesting a lack of transparency (40). Although the completion rate was higher than all trials from 2007 to 2010 (38), but reported results was significantly lower than other study (31). The possible explanation might be positive results were submitted more rapidly after completion, and studies sponsored by industries or companies were not likely to report negative results (41, 42). As a public registry platform, ClinicalTrials.gov is expected to make research more transparent and to reduce reporting bias, and sponsors are encouraged to publish their outcomes on ClinicalTrials.gov with no delay (31). Feasibility, lacking funding, unforeseen issues, poor recruitment and change project will also affect the progress of trials. In our study, 6.85% trials were suspended, terminated, or withdrawn, which was not high than previous study (38), suggesting supporting are good for such field.

In our study, a total of 37.64% trials were blinded, and 61.18% were open-labeled, the results were lower than all trials in Clinicaltrials.gov from 2007 to 2010 (38). Randomization is a hallmark of trials, and randomization with blinding can help reduce bias (43). Most trials were observational designs. Observational studies are subjected to a number of potential problems that might cause bias in the results; however, the main methodological issues can be avoided by using specific study designs (44). Therefore, more well-designed trials on AI in ED and ICU are needed to help the progress of prevention, diagnosis, and treatment of emergency and critical illness.

Trials increased a lot in the past several years. With the assistant of AI, the management of patients in ED and ICU will be greatly improved (45). In spite of advantages, we found some deficiencies of trials in this field, such as lack of results reporting, clear information losing and short of trials quantities. Thus, more efforts are needed to help registered trials in this field.

The limitations should be acknowledged. Firstly, ClinicalTrials.gov does not include all trials because some investigators and sponsors may register on other registry platforms. Secondly, our study provided only the characteristics of the registered trials. The actual strengths and weaknesses of the trials were not assessed, and some missing data may bring bias to this study. Thirdly, we did not check whether the registered trials have been published in journals. These results should be analyzed in future.

In conclusion, the current study is the first study to study registered AI trilas in ED and ICU, more trials are needed and sponsors are encouraged to report the results.

## Data Availability Statement

The raw data supporting the conclusions of this article will be made available by the authors, without undue reservation.

## Author Contributions

YZ and NL visualized the presented idea and supervised the project, YZ and GL contributed to manuscript writing, GL and YZ contributed to trial searches and preparing the manuscript draft, NL, LC, and YY revised the manuscript. All authors contributed to the article and approved the submitted version.

## Conflict of Interest

The authors declare that the research was conducted in the absence of any commercial or financial relationships that could be construed as a potential conflict of interest.
